# NAPQI adducts in patients with selective hypersensitivity to acetaminophen

**DOI:** 10.3389/fphar.2026.1726508

**Published:** 2026-01-28

**Authors:** Javier Gomez-Tabales, Jesus M. García-Menaya, Natalia Blanca-Lopez, María de las Olas Cerezo-Arias, Antonio Silva-Rodríguez, Pedro Ayuso, Elena García-Martín, José A. G. Agúndez

**Affiliations:** 1 Universidad de Extremadura, University Institute of Molecular Pathology Biomarkers, UEx, Cáceres, Spain; 2 Allergy Service, Badajoz University Hospital, Badajoz, Spain; 3 Allergy Service, Infanta Leonor University Hospital, Madrid, Spain; 4 Intensive Care Medicine Department, Badajoz University Hospital, Badajoz, Spain; 5 Facility of Innovation and Analysis in Animal Source Foodstuffs of SAIUEx, University of Extremadura, Cáceres, Spain

**Keywords:** acetaminophen, adducts, adverse drug events, biodisposition, hypersensitivity, NAPQI

## Abstract

**Introduction:**

Acetaminophen, a widely used analgesic and antipyretic, can cause adverse reactions ranging from mild urticaria to severe anaphylaxis. While interindividual differences in pharmacokinetics and genetic polymorphisms are known to affect acetaminophen metabolism, the specific mechanisms underlying hypersensitivity reactions (HSRs) remain unclear.

**Methods:**

We evaluated 28 patients with single-NSAID-induced urticaria/angioedema or anaphylaxis, but no other symptoms after acetaminophen intake. All patients demonstrated selective hypersensitivity to acetaminophen while exhibiting confirmed tolerance to acetylsalicylic acid (ASA). Oral provocation tests were conducted, and NAPQI adducts and acetaminophen metabolites were quantified in serum samples using HPLC coupled with mass spectrometry in these patients and in control individuals.

**Results:**

NAPQI generation occurred early after drug administration, within the timeframe when immediate HSRs occur. NAPQI adducts were 3-fold higher in patients with positive oral provocation compared to patients with negative oral provocation, (P = 0.028), despite lower acetaminophen doses. Detoxified NAPQI metabolites were reduced in HSR individuals, suggesting impaired detoxification. A trend toward higher adduct levels was observed in individuals with *GSTM1* null genotypes.

**Conclusion:**

Our findings indicate that NAPQI adduct generation is closely related to acetaminophen HSRs, supporting a mechanistic link between impaired NAPQI detoxification and acetaminophen HSR. Genetic variability in detoxifying enzymes, particularly GSTM1, may modulate individual susceptibility. These findings warrant further investigation into NAPQI adducts as predictive biomarkers for acetaminophen hypersensitivity.

## Introduction

Acetaminophen (paracetamol) is a drug commonly used as an over-the-counter analgesic and antipyretic properties. Although it is considered a relatively safe drug, it causes common adverse drug events that include increased levels of transaminases, hepatotoxicity, hypoglycemia, thrombopenia, hypotension, and hypersensitivity reactions (HSRs) that include different clinical entities, ranging from mild reactions such as urticaria to severe anaphylaxis. As compared to other cyclooxygenase inhibitors, acetaminophen has a narrow margin for hepatotoxicity, with recommended daily doses leading to plasma concentrations close to those related to dose-depedent toxic events.

Interestingly, acetaminophen is extensively and rapidly metabolized in humans, and it has been shown a high variability in the ability to metabolize this drug. This variability is related to interindividual differences in pharmacokinetics, and polymorphic genes coding for acetaminophen-metabolizing enzymes are determinants of acetaminophen pharmacokinetics.

The primary metabolic pathways of acetaminophen involve phase II conjugation reactions, predominantly forming acetaminophen glucuronide (approximately 52%–65% of the administered dose) and acetaminophen sulfate (around 30%–44%), (Court et al., 2017; Mazaleuskaya et al., 2015; Forrest et al., 1982). These conjugates are pharmacologically inactive and are efficiently eliminated in urine, representing the major detoxification route. However, a smaller fraction of the drug, typically 5%–10%, undergoes phase I oxidation mediated by cytochrome P450 enzymes resulting in the formation of the highly reactive intermediate N-acetyl-p-benzoquinone imine (NAPQI) (Walker et al., 2017; Leeming et al., 2017). Detoxification of NAPQI is mediated by the enzymes GSTT1, GSTM1, and GSTP1 (Coles et al., 1988), resulting in the formation of acetaminophen–glutathione conjugates, which are subsequently converted into acetaminophen–cysteine and acetaminophen–mercapturate derivatives (Hodgman and Garrard, 2012; Bessems and Vermeulen, 2001).

At therapeutic doses of acetaminophen, NAPQI is neutralized through spontaneous or enzyme-catalyzed binding to the cysteine residue of glutathione (GSH), forming an inactive metabolite known as acetaminophen-glutathione (APAP-GSH) (Court et al., 2017; Mazaleuskaya et al., 2015). This metabolite is subsequently transformed, either spontaneously or enzymatically, into acetaminophen-cysteine (APAP-Cys) and acetaminophen-mercapturate (APAP-Merc). However, if NAPQI is not metabolized, it binds to cellular proteins, leading to cellular damage, which plays a key role in acetaminophen-induced liver injury (Chidi et al., 2023).

HSRs to drugs are the third most common cause of consultations in allergy services, beta-lactams, NSAIDs, and acetaminophen being the most frequent groups of drugs involved (Dona et al., 2012), in addition to causing other adverse reactions that have led to the development of clinical practice guidelines and consensus documents for their clinical management (Ayuso et al., 2022; Theken et al., 2020). Acetaminophen is responsible for 10% of HSRs caused by NSAIDs, being one of the main drugs involved in the development of these reactions in children (Dona et al., 2012; Blanca-Lopez et al., 2015). Among the group of patients with cross-reactivity to NSAIDs, acetaminophen is involved in 12% of cases, while in patients with selective responses, acetaminophen accounts for 10% of all the reactions (Perez-Sanchez et al., 2020; Terzioglu et al., 2020).

One of the hypotheses that explains how drugs can activate the immune system and trigger hypersensitivity reactions is the hapten theory, which proposes that drugs or their metabolites covalently bind to proteins, forming immunogenic complexes (Pichler and Hausmann, 2016). This mechanism has been demonstrated with beta-lactams, and drug-protein conjugates are widely used in diagnostic tests for beta-lactam allergy (Ariza et al., 2016). For other drugs, biotransformation into reactive metabolites is necessary to enable protein binding and immunogenicity (Faulkner et al., 2014). In contrast, the p-i concept suggests that some drugs or metabolites can directly and reversibly interact with immune receptors, such as T-cell receptors or MHC molecules, without prior covalent binding or processing (Pichler, 2019).

It has been shown that at high acetaminophen doses the levels of NAPQI bound to proteins increase in parallel with the biomarker of liver toxicity. It has been demonstrated also that NAPQI adducts are detectable at therapeutic acetaminophen doses (Heard et al., 2016) and therefore it is conceivable that NAPQI-protein adducts could be involved also in acetaminophen hypersensitivity. Our *a priori* hypothesis is that some patients with selective acetaminophen hypersensitivity could exhibit increased NAPQI adduct formation and reduced detoxification compared to controls. Unfortunately, the kinetics of NAPQI generation *in vivo* at therapeutic acetaminophen doses is poorly understood, and there are no studies analyzing the generation of NAPQI in patients with HSRs to acetaminophen. To investigate the potential role of NAPQI in acetaminophen hypersensitivity, we analyzed the pharmacokinetics of NAPQI generation and detoxication *in vivo*, we determined the generation of NAPQI adducts in patients with selective HSRs to acetaminophen as compared to subjects that were not allergic, and we analyzed putative modulators of NAPQI generation and detoxication *in vivo*.

## Methods

### Patients and control subjects

This study was performed according to the principles of the Declaration of Helsinki and approved by the local ethics committee from the two Hospitals involved in the study. All patients were informed about the study, and a signed informed consent was obtained.

Subjects reporting a single NSAID-induced urticaria/angioedema or anaphylaxis, but no other symptoms after acetaminophen intake were evaluated in two centers (University Hospital, Badajoz, and Infanta Leonor University Hospital, Madrid). The study was approved by the Ethics Committees of both participating hospitals, and conducted according to the declaration of Helsinki. Twenty eight patients who reported an HSR to acetaminophen and demonstrated ASA tolerance, were included in the study groups. A challenge with acetaminophen was performed to confirm the diagnosis of single nonsteroidal anti-inflammatory drug-induced hypersensitivity (SNIUAA) (Dona et al., 2012). Eigth patients had positive oral provocation test (OPT) and were classified as SNIUAA, and 20 patients were negative after OPT. Exclussion criteria included chronic spontaneous urticaria, nasal polyposis, respiratory infections, autoimmune diseases, pregnancy, or breastfeeding. Details of patients and controls are summarized in Table 1. In addition, healthy controls who received a single IV administration of acetaminophen were included in the study group to characterize the pharmacokinetics and timing of NAPQI metabolite formation.

**TABLE 1:** Characteristics of patients and control subjects included in this study.

Characteristic	SNIUAA (n = 8)	Patients with negative oral provocation (n = 20)
Sex (F/M)	4/4	13/7
Age, years, X ± SD (range)	44.86 ± 14.31 (32–67)	47.1 ± 16.82 (19–75)
Time until sample collection, hours, X ± SD (range)	0.88 ± 0.49 (0.5–1.5)	4.5 ± 0.0 (4.5)
Cumulated APAP dose, mg, X ± SD (range)	416.7 ± 322.0 (100–850)	1,650 ± 0.0 (1,650)

Summary of sex distribution, age, time to sample collection, and cumulative acetaminophen dose in patients with selective NSAID-induced urticaria/angioedema or anaphylaxis (SNIUAA) and in subjects with negative oral provocation tests.

### Oral provocation test

OPT with acetaminophen was carried out following published recommendations (Dona et al., 2011; Perez-Alzate et al., 2016). If symptoms appeared at any time the procedure was stopped and a blood sample for determination of acetaminophen and adducts was obtained. If no response appeared, increasing doses of acetaminophen were administered until a cumulative dose of 1,500–2000 mg in 4–5 h, and after completing the procedure blood samples were collected. Clinical symptoms were assessed and changes in nasal flow and lower airway involvement were monitored by acoustic rhinomanometry and by measurement of FEV1 respectively. SNIUAA patients received lower cumulated doses of acetaminophen than control patients (Table 1), because of safety concerns during provocation.

### Analyses for acetaminophen and metabolites

Acetaminophen and its metabolites were quantified in serum samples using HPLC coupled with mass spectrometry, as previously described (Cerezo-Arias et al., 2022; Vliegenthart et al., 2017). The blood timing is shown in Table 1. The mass spectrometer operated in positive electrospray ionization mode with multiple reaction monitoring (MRM). The source conditions were set to 500 °C and 5.5 kV. The target mass-to-charge ratios (m/z) were 152→110.2, 93.2 for acetaminophen; 156→114.2, 97 for deuterated acetaminophen; 271→208, 182.2 for acetaminophen cyteine; 313→208.2, 140 for acetaminophen mercapturate; and 457→328.1, 140 for acetaminophen glutathione.

### Analyses for NAPQI adducts

NAPQI-Cys adducts were analyzed by using an Agilent 1,290 Infinity II UHPLC coupled with 6,470 triple quadrupoles (QqQ) (Agilent Technologies, Waldbronn, Germany) as described elsewhere (Cook et al., 2016). A Zorbax C18 column, 100 mm × 2.1 mm, 1.8 um (Agilent, CA) was used for the separation step working at 35 °C. The LC-MS interface was ESI with a jet stream. Nitrogen was used as nebulizing gas, drying gas, sheath gas, and collision gas. The mobile phase was formed by two solutions: A, aqueous with 0.1% formic acid, and B, acetonitrile. A binary gradient was applied with a flow rate of 0.4 mL/min: 0–1 min 2% B, 1–9 min linear increase from 2% to 90% B and kept until 10 min, followed by re-equilibration of the column until 15 min. NAPQI-Cys was eluted at 2.5 min. The ionization source parameters, operating in positive polarity, were optimized by injecting 3 mg/L of NAPQI-Cys. The best sensitivity was obtained with the following ionization source parameters: drying gas temperature at 220 °C, nebulizer at 35 psi, drying gas flow at 12 L/min, sheath gas temperature and flow rate at 350 °C and 10 L/min, respectively, and capillary voltage at 3500 V and fragmentor to 90 V. The MRM conditions were optimized by injecting the same solution at different collision energies (CE). The transitions 271 to 208 (at 5 eV CE), 271 to 140.1 (at 15 eV CE), and to 96.1 (at 23 eV CE) were the selected MRM from the point of view of sensitivity and selectivity. The quantification transitions were 271 to 208. The minimum detection level was 2.5 nM. Data processing and analysis were performed by using the MassHunter Qualitative Analysis Software (Rev B.07.00.201, Agilent Technologies, Santa Clara, CA, United States) (Heard et al., 2016; Heard et al., 2011).

### Genotyping

DNA samples were obtained from leukocytes in peripheral blood, following a standard DNA extraction protocol using the MagMAX™ Multi-Sample Ultra 2.0 DNA Kit from Thermo Fisher (ThermoFisher, Waltham, MA, United States). The analysis was conducted on the Quantum Studio 3 thermocycler. Samples were prepared by adding 1 µL of DNA (30 ng/μL) to each well, along with 6 µL of a reaction mixture composed of Master Mix, MilliQ water, and primers corresponding to each SNP. Forty cycles were performed under the following conditions: denaturation for 600 s at 95 °C, annealing for 60 s at 60 °C, and extension for 60 s at 95 °C. To quantify copy number variations (CNVs), 2 µL of DNA (5 ng/μL) was used, adding 8 µL of the reaction mixture. Determinations were carried out in triplicate. Data processing was performed using the CopyCaller software (Thermo Fisher, United States) as described elsewhere (Turner et al., 2023).

Genes coding for enzymes related to NAPQI generation and NAPQI detoxication (Agundez et al., 2018) were analyzed. We selected polymorphisms and CNVs that meet at least two of the following criteria (Court et al., 2017): minor allele frequency in the Iberian population (IBS) greater than 5% (Mazaleuskaya et al., 2015); functional effect of the polymorphisms, such as causing amino acid changes or being located in regulatory regions; and/or (Forrest et al., 1982) previously published studies supporting the functional impact of the polymorphism in the metabolism of NAPQI. Further details on the polymorphisms and CNVs analyzed, as well as the probes used, are provided in the Supplementary Table S1. Since this is an exploratory study, no formal power calculation was performed due to the rarity of selective acetaminophen hypersensitivity.

## Results

We analyzed the pharmacokinetics of acetaminophen and NAPQI metabolites in healthy individuals who received 1 mg of acetaminophen intravenously as a reference for interpreting metabolite profiles in patients. As shown in Table 2 the most abundant NAPQI metabolite was acetaminophen cysteine (Cmax = 2.94 uM), followed by acetaminophen mercapturate (Cmax = 0.56 uM). The Tmax values for these metabolites were 2.5 and 3.5 h, respectively. Interestingly, these two metabolites were already present 30 min after acetaminophen administration. Concerning acetaminophen glutathione, plasma concentrations were too low to be quantified, with the highest concentration occurring at 2.0 h. The sum of all NAPQI metabolites reached the maximum concentration 2.5 h after the administration. Free NAPQI was not detected because NAPQI is rapidly biotransformed to the above-mentioned metabolites, but these results show that NAPQI is generated early after drug administration, within the time when patients with SNIUAA develop the HSR.

**TABLE 2:** Pharmacokinetics of acetaminophen and NAPQI metabolites in healthy individuals.

Parameter	T = 0.5h	T = 1h	T = 1.5h	T = 2h	T = 2.5h	T = 3h	T = 3.5h	T = 4h	T = 4.5h	T = 5h
Acetaminophen (μM); mean ± SD (min-max)	39.60 ± 16.66 (21.77–80.50)	37.92 ± 21.37 (19.65–94.90)	34.42 ± 17.21 (18.13–77.21)	29.49 ± 17.26 (15.46–74.95)	26.27 ± 18.73 (11.47–74.51)	19.26 ± 9.19 (12.35–40.02)	16.99 ± 8.61 (8.11–39.45)	12.56 ± 8.30 (6.47–34.84)	10.47 ± 7.60 (5.89–29.79)	9.70 ± 6.05 (4.02–24.68)
Acetaminophen mercapturate (μM); mean ± SD (min-max)	0.19 ± 0.24 (0.00–0.84)	0.25 ± 0.28 (0.03–0.98)	0.39 ± 0.45 (0.08–1.51)	0.49 ± 0.50 (0.16–1.66)	0.53 ± 0.49 (0.17–1.66)	0.52 ± 0.44 (0.20–1.60)	0.56 ± 0.46 (0.18–1.51)	0.48 ± 0.38 (0.14–1.20)	0.42 ± 0.32 (0.08–0.99)	0.40 ± 0.33 (0.06–1.04)
Acetaminophen cysteine (μM); mean ± SD (min-max)	1.11 ± 2.71 (0.00–8.81)	1.45 ± 2.69 (0.09–8.80)	2.29 ± 4.01 (0.13–13.31)	2.56 ± 4.07 (0.14–13.50)	2.94 ± 4.55 (0.14–14.88)	2.91 ± 4.40 (0.14–14.51)	2.87 ± 4.19 (0.16–13.01)	2.17 ± 3.04 (0.20–10.11)	1.75 ± 2.43 (0.13–7.84)	1.77 ± 2.48 (0.17–8.24)
Acetaminophen glutathione (μM); mean ± SD (min-max)	0.00 ± 0.00 (0.00–0.00)	0.00 ± 0.00 (0.00–0.00)	0.00 ± 0.00 (0.00–0.00)	0.01 ± 0.05 (0.00–0.15)	0.00 ± 0.00 (0.00–0.00)	0.00 ± 0.00 (0.00–0.00)	0.00 ± 0.00 (0.00–0.00)	0.00 ± 0.00 (0.00–0.00)	0.00 ± 0.00 (0.00–0.00)	0.00 ± 0.00 (0.00–0.00)
Total NAPQI metabolites (μM); mean ± SD (min-max)	1.30 ± 2.94 (0.08–9.64)	1.70 ± 2.96 (0.20–9.78)	2.68 ± 4.44 (0.21–14.82)	3.05 ± 4.54 (0.30–15.10)	3.46 ± 5.02 (0.31–16.56)	3.44 ± 4.83 (0.45–16.10)	3.14 ± 4.49 (0.30–14.52)	2.65 ± 3.37 (0.36–11.20)	2.16 ± 2.72 (0.20–8.83)	2.17 ± 2.75 (0.37–9.28)

Mean plasma concentrations (μM) of acetaminophen and its major NAPQI-derived metabolites (acetaminophen mercapturate, acetaminophen cysteine, and acetaminophen glutathione) at different time points after intravenous administration. Total NAPQI, metabolite concentrations are also shown. T: time after acetaminophen administration.

We carried out an analysis of NAPQI adducts in SNIUAA patients and subjects with negative oral provocation as controls. The results, shown in Table 3, indicate major differences in NAPQI adducts between the two groups of subjects studied. Among the SNIUAA patients, all but one had detectable NAPQI adduct levels (higher than 2.5 nM), whereas only 8 out of the 20 controls had detectable adduct levels (Chi-square test P = 0.025). This finding is specially relevant considering the lower doses of acetaminophen administered to patients and the shorter mean timing of sample collection, as compared to control individuals (Table 1).

**TABLE 3:** Determination of NAPQI adducts and NAPQI metabolites in patients with SNIUAA and controls.

Parameter	SNIUAA (n = 8)	Patients with negative oral provocation (n = 20)	T-test (P-value)
NAPQI adducts (nM); mean ± SD (min-max)	27.46 ± 25.93 (0–80.82)	9.72 ± 14.33 (0–41.83)	0.028
Acetaminophen mercapturate (nM); mean ± SD (min-max)	311.5 ± 614.9 (22.0–1823.0)	1,910.6 ± 1,101.3 (20.2–3,935.9)	<0.001
Acetaminophen cysteine (nM); mean ± SD (min-max)	299.2 ± 434.1 (0–1,262.6)	1,194.0 ± 452.5 (571.0–2,652.3)	<0.001
Sum of NAPQI metabolites (nM); mean ± SD (min-max)	610.6 ± 1,031.8 (22.0–3,086.9)	2,877.2 ± 1,206.6 (1,170.0–4,770.6)	<0.001
NAPQI adducts/Sum of NAPQI metabolites; mean ± SD (min-max)	0.180 ± 0.337 (0–1,002)	0.004 ± 0.007 (0–0.25)	0.024
NAPQI adducts * 1,000/APAP concentration; mean ± SD (min-max)	1.594 ± 2.07 (0–6.34)	0.293 ± 0.45 (0–1.45)	0.011
NAPQI adducts * 1,000/Acetaminophen dose; mean ± SD (min-max)	0.059 ± 0.084 (0–0.259)	0.006 ± 0.009 (0–0.025)	0.004

Mean concentrations (nM) of NAPQI-protein adducts and major detoxified metabolites (acetaminophen mercapturate and acetaminophen cysteine), along with calculated ratios (adducts/metabolites, adducts normalized by acetaminophen concentration and dose). Statistical significance assessed by t-test.

The average adduct concentrations were nearly three-fold higher among the SNIUAA patients than in the controls, making these differences statistically significant. On the contrary, when analyzing the major NAPQI metabolites, acetaminophen mercapturate and acetaminophen cysteine, higher concentrations were detected in controls. Acetaminophen glutathione was not detectable either in SNIUAA patients or in controls. The sum of NAPQI metabolites (those that were metabolized instead of creating adducts) was five times higher in the control group. This suggests that NAPQI tends to form adducts in a greater proportion of SNIUAA patients than in subjects with negative OPT, who metabolize more efficiently NAPQI, as shown in Table 3. Although doses differed between groups, ratios normalized by dose and plasma acetaminophen concentration were calculated to minimize bias. The ratio between NAPQI adducts vs. NAPQI metabolites is also shown in Table 3, which confirms the previous hypothesis. Considering the putative effect of differences in acetaminophen doses, shown in Table 1, we calculated the ratio NAPQI adducts/acetaminophen concentration, and the ratio NAPQI adducts/total dose administered. In both cases, SNIUAA patients had higher adduct ratios than subjects with negative OPT, making the differences statistically significant. This reinforces the hypothesis that a decreased ability to metabolize NAPQI is correlated with adduct generation and that this occurs more in patients with SNIUAA.

To account for putative confounders, we determined the most common genotypes in genes coding for enzymes involved in NAPQI production and detoxication. Results, which are summarized in Table 4, do not reveal genetic factors that might significantly influence the difference in adduct concentrations between patients with SNIUAA and controls. Supplementary Table S2 contains further details of the results for every SNV and CNV analyzed independently.

**TABLE 4:** Comparison of common diplotypes for enzymes involved in NAPQI formation or degradation, and NAPQI adduct concentrations.

Diplotype	SNIUAA (n = 8); N (%)	Patients with negative oral provocation (n = 20); N (%)	NAPQI adduct concentrations; mean ± SD (T-test P value)	NAPQI adduct * 1,000/APAP concentration; mean ± SD (T-test P value)
CYP2A6 diplotype, number of non-mutated genes = 3	1	0	17.38 ± 3.28 (N.A.)	0.987 ± 0.187 (N.A.)
CYP2A6 diplotype, number of non-mutated genes = 2	5	17	16.67 ± 20.15 (reference)	0.749 ± 1.285 (reference)
CYP2A6 diplotype, number of non-mutated genes = 1	1	3	3.23 ± 2.44 (0.233)	0.10 ± 0.08 (0.378)
CYP2A6 diplotype, number of non-mutated genes = 0	1	0	16.98 ± 3.21 (N.A.)	0.75 ± 0.14 (N.A.)
CYP2D6 diplotype ultrarapid metabolizer	0	2	20.91 ± 7.90 (0.987)	0.449 ± 1.170 (0.759)
CYP2D6 diplotype normal metabolizer	3	9	20.59 ± 18.61 (reference)	0.632 ± 0.587 (reference)
CYP2D6 diplotype intermediate metabolizer	5	8	9.63 ± 9.98 (0.167)	0.780 ± 0.122 (0.788)
CYP2D6 diplotype poor metabolizer	0	1	0.00 (N.A.)	0.00 (N.A.)
GSTM1 diplotype, number of non-mutated genes = 2	0	2	0.00 (reference)	0.00 (reference)
GSTM1 diplotype, number of non-mutated genes = 1	2	8	14.29 ± 10.85 (0.501)	0.54 ± 0.40 (0.408)
GSTM1 diplotype, number of non-mutated genes = 0	6	10	16.95 ± 19.24 (0.482)	0.82 ± 1.28 (0.664)
GSTP1 diplotype, number of non-mutated genes = 2	5	12	20.97 ± 19.88 (reference)	0.92 ± 1.28 (reference)
GSTP1 diplotype, number of non-mutated genes = 1	3	8	5.23 ± 6.68 (0.228)	0.27 ± 0.32 (0.313)
GSTP1 diplotype, number of non-mutated genes = 0	0	0	—	—
GSTT1 diplotype, number of non-mutated genes = 2	4	5	13.79 ± 9.88 (reference)	0.65 ± 0.44 (reference)
GSTT1 diplotype, number of non-mutated genes = 1	3	13	15.76 ± 19.52 (0.241)	0.72 ± 1.28 (0.163)
GSTT1 diplotype, number of non-mutated genes = 0	1	2	12.64 ± 5.23 (0.520)	0.43 ± 0.18 (0.719)

Comparison of CYP2A6, CYP2D6, GSTM1, GSTP1, and GSTT1 diplotypes between SNIUAA patients and controls, with corresponding mean NAPQI adduct concentrations and normalized ratios. CYP2D6 diplotypes are reported according to the Clinical Pharmacogenomics Implementation Consortium diplotype/phenotype description (https://cpicpgx.org/guidelines/cpic-guideline-for-tamoxifen-based-on-cyp2d6-genotype/). For the rest of genes such description is not available and therefore the diplotypes are classified accoding to the number of non-mutated genes. N.A., not available. Additional details for individual SNV analyses are provided in Supplementary Table S2.

No statistically significant differences were observed between genotypes or allele frequencies in SNIUAA patients vs. controls. In addition, adduct concentrations were similar when participants were stratified according to their genotypes except for GSTM1. However, linear regression of the number of *GSTM1* copies and adduct concentrations displayed a borderline statistical significance (P = 0.050), with higher adduct concentrations in individuals with fewer *GSTM1* copies. This finding is consistent with the role of GSTM1 as a prominent NAPQI-detoxicating enzyme.

## Discussion

Growing evidence shows that drug metabolites play a prominent role in the development of drug hypersensitivity reactions DHRs. This has been already shown with beta-lactams and NSAIDs such as aspirin, diclofenac, or metamizole (Agundez et al., 2015), and metabolites of many other drugs commonly involved in DHRs could likely have a higher immunogenicity than the parent drug. Reactive drug metabolites are fastly generated after drug absorption and can trigger immune responses, as well as liver toxicity (Agundez et al., 2015; Meng et al., 2018; Blanca-Lopez et al., 2016; Tempark et al., 2022). Good examples of this are metamizole metabolites, which can increase the diagnostic potential of the basophil activation test when they are included in the assay (Ariza et al., 2016).

The role of acetaminophen metabolism in the development of hypersensitivity to this drug is poorly understood. Acetaminophen can trigger Type I-mediated hypersensitivity reactions (IgE-mediated), leading to single NSAID-induced urticaria/angioedema or anaphylaxis (SNIUAA) (Blanca-Lopez et al., 2015; Kowalski et al., 2013; Lee, 2017). Immediate HSRs to acetaminophen account for over 25% of cases (Gabrielli et al., 2018).

Since drug metabolites require protein binding to trigger HSRs (Canto et al., 2009) and because NAPQI can bind to proteins, a plausible mechanism involved in acetaminophen hypersensitivity and toxicity is oxidative metabolism that leads to the production of NAPQI (Walker et al., 2017; Jetten et al., 2016). It can be hypothesized that enhanced oxidative metabolism may result in increased bioavailability of NAPQI, which, unless quickly detoxified by GST enzymes, can bind to proteins. Therefore, increased oxidative metabolism, reduced GST metabolism, or glutathione depletion (Trettin et al., 2014) may result in heightened toxicity, and it can be speculated that this could also raise the risk of developing HSRs to acetaminophen.

This study provides novel evidence that reactive metabolite formation, specifically NAPQI adducts, may contribute to HSRs to acetaminophen. Elevated adduct levels in hypersensitive patients, despite lower drug exposure, suggest a reduced capacity for NAPQI detoxification. This aligns with the hypothesis that reactive metabolites can act as haptens, triggering immune responses. Importantly, NAPQI metabolites were detectable within 30 min of drug administration (Table 2), coinciding with the typical onset of immediate hypersensitivity reactions. This temporal alignment strengthens the case for a causal role of NAPQI in these events.

We have further demonstrated that the gross values of NAPQI adducts, along with the ratios of adducts/acetaminophen and adducts/dose, are significantly elevated in patients with SNIUAA compared to controls. Heard et al. also detected adducts after five doses in most healthy subjects (Heard et al., 2016). In contrast, our findings demonstrate that a single administration is enough to generate NAPQI adducts in most SNIUAA patients.

Moreover, the concentration of detoxified NAPQI metabolites is reduced in SNIUAA patients. These results are in line with the hypothesis that patients with SNIUAA have a diminished capacity to detoxify NAPQI, leading to increased bioavailable NAPQI that forms adducts. These findings provide strong support for the role of NAPQI-protein binding in acetaminophen HSRs. Additional implications of this variability could affect the metabolism of other drugs: Acetaminophen is a known inhibitor of NAT2 (Rothen et al., 1998; Tahir et al., 2016), and variability in reactive metabolite formation may influence this effect. Such inhibition can alter NAT2 activity prior to phenotyping, potentially explaining inconsistencies between genotype and phenotype associations (Kotila et al., 2019; Straka et al., 2006; Taja-Chayeb et al., 2011) and disease studies (Evans, 1984; el-Yazigi et al., 1992; Irsh et al., 1992; Agundez et al., 1996; Hegele et al., 2000). This mechanism is currently under investigation in our laboratory.

The role of polymorphic drug metabolism in the ability to synthesize and detoxify NAPQI in its bioavailability could be a modifier of risk. Among the genes involved in the formation of NAPQI, *CYP1A2, CYP2A6, CYP2D6, CYP2E1*, and *CYP3A4*, we analzyed common gene variations in the genes *CYP1A2, CYP2A6*, and *CYP2D6*, but not for *CYP2E1* or *CYP3A4* because of the absence of common gene variations with a clear functional impact in the population studied (with a minor allele frequency higher than 0.05) and, therefore, even in the event of detecting an individual with these rare SNVs, any statistical comparison would yield non-significant results.

We did not find statistically significant differences when comparing the genotypes of enzymes related to NAPQI, but this could be due to the sample size and the relatively low minor allele frequencies for many of the gene variants analyzed. A larger series of patients developing SNIUAA with acetaminophen is required to demonstrate a genetic predisposition. Interestingly, NAPQI adducts were higher in individuals with genetically determined impaired GSTM1 activity as a consequence of complete gene deletion, thus reinforcing the hypothesis that a decreased ability to detoxify NAPQI could lead to increased formation of adducts.

To date, no studies have examined the potential impact of genetic variations on the risk of developing acetaminophen HSRs or its clinical presentation. Research on GST enzymes has demonstrated that acetaminophen exposure during infancy, in combination with the common *GSTP1* single nucleotide variation rs1695, increases the risk of developing asthma (Kang et al., 2013). In the same way, prenatal exposure to acetaminophen, coupled with *GSTM1* and *GSTT1* polymorphisms, has been shown to modify this risk (Perzanowski et al., 2010), although some findings remain still controversial (Shaheen et al., 2010; Riley et al., 2015). In contrast, the influence of these genetic variations on acetaminophen HSRs in adults needs to be investigated.

According to our findings, reduced GSTM1 activity is linked to increased NAPQI adduct formation, making plausible that individuals with null GSTM1 genotypes—present in approximately half of the Spanish population (Lucena et al., 2008) —may be at higher risk of developing acetaminophen HSRs. Notably, our data indicates that 6 out of 8 SNIUAA patients (75%) carry the *GSTM1* null genotype, compared to 50% in the control group. Although this difference is not statistically significant due to the small sample size, it highlights the necessity of further research. No differences were observed in allele frequencies for *GSTT1* or *GSTP1*. Previous studies support a stronger association of *GSTM1* null genotypes, compared to *GSTT1* null genotypes, with idiosyncratic drug-induced liver injury (Lucena et al., 2008).

The potential role of GSTM1 in DHRs may also be linked to its involvement in oxidative stress (Ayuso et al., 2021). Further studies with larger cohorts of patients experiencing acetaminophen HSRs are needed to comprehensively investigate the influence of polymorphisms in GSTM1, GSTT1, GSTP1, and other enzymes involved in redox regulation, which have been associated with drug-induced liver injury (Lucena et al., 2008; Lucena et al., 2010; Sun et al., 2020; Agundez et al., 2011; Andrade et al., 2009).

Although this study provides preliminary evidence supporting a mechanistic link between NAPQI adduct formation and selective hypersensitivity reactions to acetaminophen, several limitations should be acknowledged. First, the sample size is small, particularly in the HSR group (n = 8), which limits statistical power and generalizability. While the study design aligns with the scope of case series contributions in this Research Topic (detailed case studies that provide a blueprint for integrating genetic data into patient care), larger cohorts are needed to confirm the observed trends and strengthen genotype–phenotype associations. Second, there were notable differences in acetaminophen dosing and timing of sample collection between HSR patients and controls. Although normalization strategies were applied, these variables may still confound comparisons of metabolite and adduct levels. Future studies should aim for standardized dosing and sampling protocols across groups. Third, while genotyping was performed for key enzymes involved in NAPQI metabolism, the absence of functional validation limits mechanistic insight. Nevertheless, it should be stated that all tested variants have well known functional effects. The observed association with GSTM1 null genotypes, although biologically plausible, did not reach statistical significance and requires replication in larger, independent cohorts. Additionally, since all participants were recruited from specialized allergy centers, there is a possibility of selection bias, which may limit the generalizability of the findings to broader clinical populations. Finally, the study focused exclusively on adult patients and did not explore potential age-related differences in acetaminophen metabolism or hypersensitivity risk. Pediatric populations, where acetaminophen use is frequent, may exhibit distinct metabolic or immunological profiles. Also, acetaminophen metabolism may be different in elderly and pregnant women, which may influence metabolite profiles and warrants future research. Despite these limitations, the findings offer a valuable proof-of-concept and highlight the need for further research into the role of reactive metabolites in drug hypersensitivity reactions.

In summary, our results show that reactive metabolites such as NAPQI are rapidly generated following acetaminophen administration and that patients with acetaminophen HSRs exhibit increased NAPQI adducts and decreased detoxified NAPQI metabolites. These findings provide proof of concept for the role of NAPQI adducts in immediate reactions to acetaminophen and highlight the potential influence of genetic variability in the enzymes responsible for NAPQI generation and detoxification as modifiers of the response to acetaminophen. These findings also suggest a potential role for reactive drug metabolites in DHRs that deserve further research.

## Data Availability

The original contributions presented in the study are included in the article/Supplementary Material, further inquiries can be directed to the corresponding authors.
