# Supplementary material for: NAPQI adducts in patients with selective hypersensitivity to acetaminophen
Source: Front Pharmacol. 2026 Jan 28;17:1726508. doi: 10.3389/fphar.2026.1726508 (PMC12892134; doi:10.3389/fphar.2026.1726508)
Supplement: Supplementary file 1 [file DataSheet1.pdf]

NAPQI adducts in patients with selective hypersensitivity to acetaminophen.

Running title: *NAPQI adducts in hypersensitivity to acetaminophen*

Javier Gomez-Tabales [1]*, Jesus M García-Menaya [1,2]*, Natalia Blanca-Lopez [3], María de las Olas Cerezo-Arias [1,4], Antonio Silva-Rodríguez [5], Pedro Ayuso [1], Elena García-Martín [1] ‡ and José A. G. Agúndez [1] ‡

Supplemental Table 1: Single nucleotide variations (SNVs) and copy number variations (CNVs) analyzed in this study.

Gene	Variation	Variant Id.	Probe Id.	Location (GRCh38)	Effect
CYP1A2	SNV	rs762551	C_8881221	15-74749576-C-A	intron
CYP2A6	SNV	rs1801272	C_27861808	19-40848628-A-T	missense
CYP2A6	SNV	rs28399433	C_30634332	19-40850474-A-C	intron
CYP2A6	CNV	nsv1065886	Hs07545274_cn	19-40850173	deletion
CYP2D6	SNV	rs1065852	C__11484460	22-42130692-G-A	missense
CYP2D6	SNV	rs3892097	C_27102431	22-42128945-C-T	splice acceptor
CYP2D6	SNV	rs35742686	C_32407232	22-42128241-CT-C	frameshift
CYP2D6	SNV	rs16947	C__27102425	22-42127941-G-A	missense
CYP2D6	SNV	rs28371725	C_34816116	22-42127803-C-T	intron
CYP2D6	SNV	rs1135840	C__27102414	22-42126611-C-G	missense
CYP2D6	CNV	nsv3642	Hs00010001_cn	22:42126649	deletion
GSTM1	SNV	rs1065411	C_44202997	1-109690516-G-C	missense
GSTM1	CNV	nsv276	Hs02595872_cn	1-109689314	deletion
GSTP1	SNV	rs1695	C_3237198	11-67585218-A-G	missense
GSTP1	SNV	rs1138272	C_1049615	11-67586108-C-T	missense
GSTT1	CNV	nsv436358	Hs00659429_cn	22:270308	deletion

Supplemental Table 2:

Association between individual SNVs and CNVs and NAPQI adduct concentrations.

Genetic factor	SNIUAA (n=8); N (%)	Patients with negative oral provocation (n=20); N (%)	NAPQI adduct concentrations; mean ± SD (T-test P value)	NAPQI adduct * 1000 / APAP concentration; mean ± SD (T-test P value)
CYP1A2 rs762551 A/A	5	11	11.43 ± 11.83 (reference)	0.424 ± 0.411 (reference)
CYP1A2 rs762551 A/C	2	7	19.61 ± 17.94 (0.378)	0.565 ± 0.556 (0.608)
CYP1A2 rs762551 C/C	1	2	18.22 ± 7.82 (0.472)	2.255 ± 1.199 (0.038)
CYP2A6 rs1801272 A/A	7	20	14.71 ± 19.82 (reference)	0.66 ± 1.27 (reference)
CYP2A6 rs1801272 A/T	0	0	--	--
CYP2A6 rs1801272 T/T	1	1	16.99 ± 3.21 (N.A.)	0.75 ± 0.14 (N.A.)
CYP2A6 rs28399433 A/A	7	17	16.72 ± 19.83 (reference)	0.76 ± 1.28 (reference)
CYP2A6 rs28399433 A/C	1	3	3.234 ± 2.444 (0.209)	0.102 ± 0.077 (0.348)
CYP2A6 rs28399433 C/C	0	0	--	--
CYP2A6 CNV 3 copies	1	0	17.38 ± 3.28 (N.A.)	0.987 ± 0.186 (N.A.)
CYP2A6 CNV 2 copies	7	19	14.30 ± 19.88 (reference)	0.644 ± 1.279 (reference)
CYP2A6 CNV 1 copy	0	1	24.99 ± 4.72 (N.A.)	0.886 ± 0.167 (N.A.)

CYP2A6 diplotype, number of non-mutated genes = 3	1	0	17.38 ± 3.28 (N.A.)	0.987 ± 0.187 (N.A.)
CYP2A6 diplotype, number of non-mutated genes = 2	5	17	16.67 ± 20.15 (reference)	0.749 ± 1.285 (reference)
CYP2A6 diplotype, number of non-mutated genes = 1	1	3	3.23 ± 2.44 (0.233)	0.10 ± 0.08 (0.378)
CYP2A6 diplotype, number of non-mutated genes = 0	1	0	16.98 ± 3.21 (N.A.)	0.75 ± 0.14 (N.A.)
CYP2D6 rs1065852 G/G	2	12	19.28 ± 18.77 (reference)	0.553 ± 0.558
CYP2D6 rs1065852 G/A	6	7	11.09 ± 11.66 (0.291)	0.838 ± 1.235 (0.575)
CYP2D6 rs1065852 A/A	0	1	0.00	0.00
CYP2D6 rs3892097 C/C	3	13	20.95 ± 19.67 (reference)	0.598 ± 0.594 (reference)
CYP2D6 rs3892097 C/T	5	6	7.18 ± 8.28 (0.075)	0.823 ± 1.221 (0.665)
CYP2D6 rs3892097 T/T	0	1	0.00	0.00
CYP2D6 rs35742686 T/T	8	18	14.16 ± 19.74 (reference)	0.675 ± 1.281 (reference)
CYP2D6 rs35742686 T/-	0	2	23.11 ± 6.38 (0.544)	0.546 ± 0.184 (0.893)
CYP2D6 rs35742686 -/-	0	0	--	--
CYP2D6 rs16947 G/G	5	11	9.42 ± 12.14 (reference)	0.596 ± 1.213 (reference)
CYP2D6 rs16947 G/A	3	6	18.96 ± 16.81 (0.254)	0.652 ± 0.548 (0.922)
CYP2D6 rs16947 A/A	0	3	30.94 ± 10.11 (0.031)	1.071 ± 0.349 (0.618)
CYP2D6 rs28371725 C/C	7	17	9.91 ± 13.31 (reference)	0.559 ± 1.227 (reference)
CYP2D6 rs28371725 C/T	1	3	44.08 ± 17.87	1.301 ± 0.541

CYP2D6 rs28371725 T/T	0	0	--	--
CYP2D6 rs1135840 G/G	1	5	18.37 ± 10.43 (reference)	0.700 ± 0.385 (reference)
CYP2D6 rs1135840 G/C	7	10	15.16 ± 18.87 (0.753)	0.784 ± 1.282 (0.901)
CYP2D6 rs1135840 C/C	0	5	9.24 ± 6.38 (0.349)	0.218 ± 0.147 (0.133)
CYP2D6 CNV 4 copies	0	1	0.00	0.00
CYP2D6 CNV 3 copies	0	1	41.82 ± 7.90 (N.A.)	0.898 ± 0.169 (N.A.)
CYP2D6 CNV 2 copies	8	18	14.32 ± 19.08 (reference)	0.682 ± 0.128 (reference)
CYP2D6 CNV 1 copy	0	0	--	--
CYP2D6 CNV 0 copies	0	0	--	--
CYP2D6 diplotype ultrarapid metabolizer	0	2	20.91 ± 7.90 (0.987)	0.449 ± 1.170 (0.759)
CYP2D6 diplotype normal metabolizer	3	9	20.59 ± 18.61 (reference)	0.632 ± 0.587 (reference)
CYP2D6 diplotype intermediate metabolizer	5	8	9.63 ± 9.98 (0.167)	0.780 ± 0.122 (0.788)
CYP2D6 diplotype poor metabolizer	0	1	0.00 (N.A.)	0.00 (N.A.)
GSTM1 rs rs1065411G/G	0	4	11.92 ± 10.85 (reference)	0.45 ± 0.40 (reference)
GSTM1 rs rs1065411 G/C	0	0	--	--
GSTM1 rs rs1065411 C/C	2	10	0.00	0.00
GSTM1 CNV 2 copies	0	2	0.00 (reference)	0.00 (reference)
GSTM1 CNV 1 copy	2	12	10.21 ± 10.85 (0.553)	0.38 ± 0.40 (0.472)

GSTM1 CNV 0 copies	6	6	22.59 ± 19.24 (0.432)	1.11 ± 1.28 (0.626)
GSTM1 diplotype, number of non-mutated genes = 2	0	2	0.00 (reference)	0.00 (reference)
GSTM1 diplotype, number of non-mutated genes = 1	2	8	14.29 ± 10.85 (0.501)	0.54 ± 0.40 (0.408)
GSTM1 diplotype, number of non-mutated genes = 0	6	10	16.95 ± 19.24 (0.482)	0.82 ± 1.28 (0.664)
GSTP1 rs1695 A/A	5	12	20.97 ± 19.88 (reference)	0.92 ± 1.28 (reference)
GSTP1 rs1695 A/G	3	8	5.23 ± 6.68 (0.223)	0.27 ± 0.32 (0.313)
GSTP1 rs1695 G/G	0	0	--	--
GSTP1 rs1138272 C/C	8	19	15.34 ± 19.63 (reference)	0.69 ± 1.27 (reference)
GSTP1 rs1138272 C/T	0	1	0.00	0.00
GSTP1 rs1138272 T/T	0	0	--	--
GSTP1 diplotype, number of non-mutated genes = 2	5	12	20.97 ± 19.88 (reference)	0.92 ± 1.28 (reference)
GSTP1 diplotype, number of non-mutated genes = 1	3	8	5.23 ± 6.68 (0.228)	0.27 ± 0.32 (0.313)
GSTP1 diplotype, number of non-mutated genes = 0	0	0	--	--
GSTT1 CNV	4	5	13.79 ± 9.88 (reference)	0.65 ± 0.44 (reference)
GSTT1 CNV	3	13	15.76 ± 19.52 (0.241)	0.72 ± 1.28 (0.163)
GSTT1 CNV	1	2	12.64 ± 5.23 (0.520)	0.43 ± 0.18 (0.719)

Comparison of genotype frequencies in SNIUAA patients and controls, with corresponding mean NAPQI adduct concentrations and normalized ratios (adducts × 1000 / acetaminophen concentration). Statistical significance assessed by t-test.
